# Live interactive singing in live streams and loneliness among international students: social connection, social support and FoMO

**DOI:** 10.3389/fpsyg.2026.1848596

**Published:** 2026-06-03

**Authors:** Yichao Lin, Xin Wang

**Affiliations:** 1 College of Arts, Xiamen University, Xiamen, Fujian, China; 2 School of Journalism and Communication, Chongqing University, Chongqing, China

**Keywords:** fear of missing out, international students, live streaming, loneliness, perceived social support, singing, social connection

## Abstract

**Background:**

International students are especially vulnerable to loneliness because geographic displacement can disrupt social ties, reduce access to familiar cultural environments, and increase acculturative stress. Although active online participation has been linked to better psychosocial outcomes, less is known about whether a specific live-stream behavior, live interactive singing, is associated with lower loneliness among international students.

**Methods:**

A cross-sectional online survey was conducted with 685 international students who used native-language live-streaming platforms and had participated in at least four live interactive singing session in the past month. Self-report measures assessed live interactive singing behavior, social connection, perceived social support, fear of missing out, and loneliness. A moderated serial mediation model was tested while controlling for gender, academic level, and offline social-life quality.

**Results:**

Live interactive singing behavior was negatively associated with loneliness. This association was statistically consistent with indirect pathways through social connection and perceived social support, including a significant serial indirect pathway through both mediators. Fear of missing out moderated the associations between live interactive singing behavior and the two mediators, as well as its direct association with loneliness, such that the beneficial pattern was weaker at higher levels of fear of missing out.

**Conclusion:**

Live interactive singing was associated with lower loneliness among international students. Because the design is cross-sectional, the observed relationships are correlational, and causal direction cannot be inferred. Theoretically, this study sharpens research on loneliness by showing the importance of specifying concrete forms of online participation and situating them within broader relational and motivational frameworks. Practically, it highlights the relevance of culturally familiar interactive digital environments as supportive spaces for international students and provides a foundation for future student-support and platform-based efforts to address loneliness in cross-cultural contexts.

## 1 Introduction

International students are among the most psychologically vulnerable populations in higher education. The experience of studying abroad simultaneously disrupts multiple pillars of social life: separation from family and close friends, immersion in a foreign linguistic environment that constrains spontaneous communication, and confrontation with unfamiliar cultural norms that render everyday social scripts ineffective (Ammigan et al., 2022; Kulari et al., 2025; Tang et al., 2025). These co-occurring stressors produce acculturative stress, a constellation of anxiety, disorientation, and identity confusion that is consistently associated with elevated loneliness (Berry, 2005; Jarrar and Nweke, 2025; Koo et al., 2021). Loneliness, defined as the aversive state arising from a perceived discrepancy between actual and desired social relationships (Peplau and Perlman, 1982), is associated with well-documented negative outcomes: it is prospectively associated with depression, anxiety, cognitive decline, impaired immune function, and increased mortality risk (Hawkley and Cacioppo, 2010; Holt-Lunstad et al., 2015; Park et al., 2020). For international students, the problem is compounded by the structural nature of their social deficit. Extremely high loneliness rates have been documented globally, reaching up to 72% in the United Kingdom (Wawera and McCamley, 2020) and between 60 and 65% in Australia (Maharaj et al., 2025), and foundational evidence indicates that loneliness emerges early in the sojourn and persists rather than dissipating with time (Sawir et al., 2008). Unlike loneliness arising from dispositional factors such as social anxiety or introversion (O’Day and Heimberg, 2021), their isolation is primarily situational, imposed by geographic displacement rather than by psychological inhibition, making it, in principle, amenable to intervention through accessible social interaction opportunities.

When social needs are unmet in the immediate environment, individuals characteristically seek alternative avenues of connection. The social compensation hypothesis posits that those who are structurally disadvantaged in offline social contexts, whether due to geographic isolation, social anxiety, or cultural displacement, turn to mediated channels to fulfil belonging needs that cannot be satisfied face-to-face (Akbari et al., 2021; O’Day and Heimberg, 2021). For international students, this compensatory impulse takes a culturally specific form: rather than seeking connection within the host society, where language barriers and cultural distance remain formidable obstacles, they gravitate toward their home culture, reconnecting with native-language communities, familiar cultural practices, and home-country social circles (Kianpour et al., 2025; Tang et al., 2025). Research on expatriates and digital nomads is consistent with this pattern: the initial stages of cross-cultural relocation are marked by intensive use of digital technologies to maintain ties with the home culture and to seek out culturally congruent social environments online (Jarrar and Nweke, 2025; Miguel et al., 2025). Digital communication thus becomes not merely a convenience but a significant resource, the primary practical means through which displaced individuals attempt to restore the social belonging that physical relocation has disrupted.

Yet not all forms of digital communication are equally associated with lower loneliness. A large and growing body of research has examined the relationship between social media use and psychological wellbeing, and the central lesson of this literature is that the mode of engagement matters far more than the mere fact of being online. The now-dominant active–passive use framework proposes that active engagement, direct, reciprocal exchange such as commenting, messaging, and responding, is associated with better outcomes, while passive consumption, browsing and scrolling without interaction, tends to correlate with poorer outcomes (Burke et al., 2011; Verduyn et al., 2017). However, meta-analytic evidence has revealed that most effect sizes for both forms are negligible, suggesting that the active–passive dichotomy is too coarse to capture the psychological dynamics at play (Godard and Holtzman, 2024). Indeed, recent literature argues that the psychological benefits of active use depend heavily on the presence of real-time reciprocity and contextual warmth (Ozimek et al., 2023; Verduyn et al., 2022). A more precise criterion comes from research on mediated social interaction: only synchronous, directed, one-on-one exchanges are associated with feelings of relatedness and are perceived by users as genuine social interaction; broadcasting, re-posting, browsing, and one-click acknowledgments do not meet this threshold (Hall, 2018). This specificity raises a critical question for intervention: if loneliness reduction requires not just active use but a particular kind of active use, synchronous, directed, and high in social presence, then which digital environments best support such interaction? Emerging research points to chat-based live streaming as a prime candidate, where high temporal proximity and “cyber-social relations” foster immediate reciprocal exchanges and robust social presence (Scheibe et al., 2022).

Live streaming has emerged as a digital environment that is well-positioned to support this kind of interaction. Unlike asynchronous social media platforms, live streaming creates a shared temporal frame in which streamers and viewers co-exist simultaneously, enabling real-time, multi-modal exchange that fosters unique “cyber-social relations” (Scheibe et al., 2022; Xue et al., 2020). To date, the live-streaming literature has predominantly examined this medium through a commercial lens, focusing on platform stickiness, viewer loyalty, purchase intention, and virtual gift-giving as outcome variables (Hu and Chaudhry, 2020; Johnson and Woodcock, 2019; Xi et al., 2023). A smaller but growing body of work has begun to investigate the psychological and relational dimensions of live-stream participation, suggesting that active engagement is associated with higher social presence, social connectedness, and subjective wellbeing (Goh and Tandoc, 2024; Onderdijk et al., 2021; Wolff and Shen, 2024). Specifically, synchronous live interactions have been characterized as a “social surrogate,” in which feelings of shared agency and social presence are associated with lower loneliness during periods of physical and social isolation (Onderdijk et al., 2021). However, this psychological line of inquiry has focused almost exclusively on general adult populations within domestic media contexts. The application of live-streaming research to cross-culturally displaced populations, for whom the medium may serve a fundamentally different psychological function—namely, the reconstitution of culturally congruent belonging—remains virtually unexplored. Recent scoping work consolidates this fragmentation: live-streaming research is dominated by gaming, esports, and e-commerce, while non-gaming social genres—including music and singing-centric streams—remain comparatively underexamined despite their rapid growth in Asian platform ecosystems (Pagáč et al., 2025). Behavioral research on Chinese live-streaming users further documents that engagement is heterogeneous in form; watching, gifting, chatting, and co-performing constitute structurally distinct modes with different relational implications (Lu et al., 2018). Recent narrative work highlights that international students in host countries often face persistent offline isolation, characterized by limited friendships with domestic peers and underutilized institutional supports (Ivanova et al., 2025). This structural offline deficit fundamentally drives displaced individuals toward home-language digital environments and online diasporic networks as their primary outlets for emotional and social support (Kianpour et al., 2025; Tang et al., 2025).

Within the live-streaming ecosystem, chat-based streaming, characterized by low participation barriers, unstructured conversation, and emotional companionship (Yang et al., 2023), offers a particularly relevant context for studying loneliness among displaced populations. Among its interactive features, live interactive singing—often conceptualized as a form of “co-performing” (Lu et al., 2018)—represents an especially compelling focal behavior. Live interactive singing requires real-time vocal coordination between a viewer and a streamer, conveys emotional tone and cultural identity through the embodied act of vocal performance, and represents one form of the synchronous, directed interaction the literature identifies as relationally consequential (Hall, 2018). For international students accessing native-language platforms, live interactive singing may function as a form of digital homecoming: a temporary re-entry into the sonic and emotional landscape of the home culture that satisfies belonging needs in ways that text-based or emoji-based interactions cannot. Experimental evidence is consistent with this characterization: in a randomized controlled trial, online group singing was associated with larger reductions in loneliness and greater increases in social participation than verbal exchange alone, suggesting that the embodied vocal element rather than shared activity alone may underlie the relational benefit (Schäfer, 2023). This leads to the central question of the present study: h*ow can live interactive singing in live streams be associated with lower loneliness among international students, and through what psychological mechanisms and under what conditions?*

Answering this question requires specifying not only whether live interactive singing is associated with lower loneliness but how. The social media–loneliness literature has identified two relational constructs as key mediating mechanisms. Social connection, the subjective perception of belonging and closeness to others (Grieve et al., 2013; Seppala et al., 2013), captures the immediate psychological yield of a positive social interaction: the felt sense that one is part of a social world. Perceived social support, the stable appraisal that emotional, instrumental, and informational resources are available from one’s network (Wills and Shinar, 2000), reflects a more enduring cognitive evaluation that develops as interactional experiences accumulate. Both have been independently shown to mediate the relationship between social media communication and loneliness (Heo et al., 2015; Zhang et al., 2021), and social support in particular emerges as the outcome most robustly associated with active social media use across over 140 studies (Godard and Holtzman, 2024; Meier and Reinecke, 2021). Yet existing models typically treat these constructs as parallel mediators, overlooking the possibility, supported by social presence theory and social capital theory, that they represent sequential stages of a single process within immersive digital environments: situational experiences of connection must first accumulate before they develop into stable perceptions of available support (Biocca et al., 2003; Lin, 1999). Disentangling this sequence is critical for understanding not just whether digital engagement helps, but at which stage of the psychological process the benefits are generated, and where the process may break down. Recent work on international students in China is consistent with this sequential framing: among 530 international students, social connectedness was indirectly associated with psychological wellbeing through loneliness and perceived stress, and perceived social support functioned specifically as a moderating resource that operated downstream of more immediate relational experiences (Tang et al., 2025; see also Razgulin et al., 2023).

This last point raises an equally important question: when does live interactive singing fail to be associated with lower loneliness? The psychological benefits of active digital participation are not unconditional; their realization depends on the motivational quality of engagement. Self-determination theory provides the relevant framework: behaviors that satisfy the needs for relatedness, competence, and autonomy yield wellbeing benefits, whereas the same behaviors performed under extrinsic, anxiety-driven regulation do not (Deci et al., 2017; Ryan and Deci, 2000). Fear of missing out (FoMO; Przybylski et al., 2013) indexes precisely this anxiety-driven motivational mode. Driven by the FoMO, individuals frequently engage in compulsive digital monitoring rather than seeking autonomous, meaningful social connection (Putri et al., 2026; Gugushvili et al., 2020).

Furthermore, FoMO is theoretically and empirically distinct from social comparison: although the two are correlated, comparison concerns evaluative judgments about one’s relative standing, whereas FoMO concerns anxious vigilance about missed opportunities for inclusion (Tandon et al., 2021). Consequently, when driven by FoMO, the potentially restorative act of live interactive singing may degrade into an anxious obligation, undermining its capacity to alleviate loneliness. Recent meta-analytic and review evidence establishes FoMO as a robust boundary condition on digital engagement outcomes, attenuating the relational yield of platform participation under conditions of stress and uncertainty (Akbari et al., 2021; Putri et al., 2026). For international students specifically, separation from home-country social circles may make FoMO particularly potent, because the platform displays the very social world from which they are absent.

The present study addresses these questions by testing a moderated serial mediation model (PROCESS Model 85; Hayes et al., 2022) in a sample of 685 international students. This study aims to make three contributions. First, it offers a theoretical refinement of the active use framework by examining a specific, functionally defined digital behavior, live interactive singing, rather than treating active use as a monolithic construct, and by identifying fear of missing out as a boundary condition that may weaken the associations between participation and relational outcomes. Second, it proposes and tests a sequential mediation in which social connection and perceived social support are positioned as ordered stages, potentially clarifying the process through which digital engagement relates to loneliness. Third, from a practical standpoint, this study explores whether live interactive singing on native-language platforms may represent an accessible, low-cost relational resource for international students, while also identifying the conditions under which this resource may be less effective, providing actionable guidance for support services and platform designers.

## 2 Hypotheses development

### 2.1 Loneliness among international students

International students are a high-risk population for loneliness (Ivanova et al., 2025). The experience of studying abroad involves simultaneous disruption across multiple social dimensions: separation from family, close friends, and familiar community structures; immersion in a foreign linguistic environment that constrains spontaneous communication; and confrontation with cultural norms and social practices that differ from those internalized during socialization (Ivanova et al., 2025; Smith and Khawaja, 2011). These co-occurring stressors produce acculturative stress, a constellation of psychological difficulties arising from the process of adapting to a new cultural context (Berry, 2005; Jarrar and Nweke, 2025). Consequently, the initial stages of cross-cultural relocation are particularly marked by social isolation, disorientation, and loneliness, as existing social capital becomes geographically inaccessible while new social networks have not yet formed (Caligiuri and Lazarova, 2002; Koo et al., 2021).

Loneliness, the aversive subjective state arising from a perceived discrepancy between actual and desired social relationships (Peplau and Perlman, 1982), can be further differentiated into two forms relevant to the international student experience. Emotional loneliness pertains to the absence of an intimate attachment figure, while social loneliness refers to a deficiency in one’s broader social network (Deckx et al., 2018). International students are simultaneously vulnerable to both: geographic separation from intimate others produces emotional loneliness, while the difficulty of forming friendships in a culturally unfamiliar environment produces social loneliness. Importantly, loneliness is driven by perceived rather than objective social deficits; an individual may be surrounded by peers and still experience profound loneliness if the quality of interactions falls short of relational expectations (Hawkley and Cacioppo, 2010). This cognitive discrepancy perspective is particularly salient for international students, who may have superficial access to peers in academic settings but lack the culturally congruent, emotionally resonant connections that satisfy deeper belonging needs (Arkoudis et al., 2019; Ivanova et al., 2025).

Faced with these relational deficits, international students characteristically turn to their home culture for psychological comfort. This digital return takes multiple forms: maintaining contact with family through messaging applications, consuming native-language media, and participating in online communities organized around shared cultural identity (Kianpour et al., 2025; Tang et al., 2025). The broader literature on mobile and displaced populations supports this pattern. Digital nomads, another group characterized by geographic dislocation and transient networks, rely heavily on digital platforms to “anchor” their liquid lifestyles, using social media both to maintain existing relationships (bonding social capital) and to establish new connections with like-minded individuals (bridging social capital; Aufschnaiter et al., 2021; Miguel et al., 2025). However, this research also reveals that the mere use of digital platforms does not guarantee loneliness reduction. When digital interactions remain superficial or are pursued under conditions of anxiety, they may reinforce rather than alleviate perceived loneliness, producing “social burnout” (Miguel et al., 2025). The medium, the mode of engagement, and the psychological state of the user jointly determine whether online communication serves as a genuine social resource or merely an anxious reflex.

Among the home-culture digital activities that international students from China characteristically choose, live interactive singing has emerged as a behaviorally common practice. Native-language live-streaming platforms—such as Bilibili Live, NetEase CC, KuGou Live, and the live channels of QQ Music—host music and singing rooms as a major content category alongside gaming and shopping streams. The heterogeneity of engagement modes within these platforms (watching, gifting, chatting, and co-performing; Lu et al., 2018) means that singing rooms attract not only spectators but a substantial population of vocal co-participants. Recent narrative work with Chinese international students in the United States and Japan documents that limited friendships with domestic peers and underutilized institutional supports create a persistent offline social deficit (Ivanova et al., 2025), which structurally drives these students toward home-language digital environments as their primary social outlet (Tang et al., 2025). Furthermore, recent empirical evidence on Chinese live-streaming ecosystems demonstrates that singing and talent-centric streams occupy a distinctive and highly popular role within users’ everyday platform repertoires, effectively satisfying their affective and social integrative needs through accessible cultural-musical content and low participation barriers (Mao, 2022). Live interactive singing is therefore not a marginal use case but a behaviorally typical avenue through which Mainland Chinese students abroad re-enter their home-cultural soundscape, justifying its selection as the focal behavior of the present study.

### 2.2 Interactive singing in live streaming

Among the digital avenues through which international students seek connection, live streaming occupies a distinctive position. As noted in Section 1, live streaming creates a shared temporal frame that approximates the immediacy of face-to-face communication. The mediated social interaction literature identifies synchrony, social presence, and reach as the three dimensions along which digital communication varies, positioning synchronous, high-social-presence, low-reach interactions as those most likely to constitute genuine social interaction (Baym, 2010; Hall, 2018). Chat-based live streaming satisfies these criteria: it features real-time dialogue, conveys social cues through vocal tone and emotional expression, and—in the case of live interactive singing—involves directed exchange and “co-performing” between identifiable individuals (Lu et al., 2018; Yang et al., 2023).

Recent scoping work nonetheless reveals that the live-streaming literature remains heavily skewed toward gaming, esports, and e-commerce, while non-gaming social genres including music and singing-centric streams remain underexamined despite their rapid growth in Asian platform ecosystems (Pagáč et al., 2025). Two generations of social-media-and-loneliness research illustrate why this granularity matters. The first examined the overall relationship between social media use and loneliness, concluding that loneliness is a risk factor for problematic use but that the reverse pathway has received far less support (O’Day and Heimberg, 2021). The second introduced the active–passive distinction (Verduyn et al., 2017); however, meta-analytic evidence is consistent with the finding that most effect sizes for both forms fall below practical significance thresholds (Godard and Holtzman, 2024; Valkenburg et al., 2022). These mixed findings have prompted calls for specifying which forms of active use, for which populations, and under what conditions produce meaningful outcomes (Meier and Krause, 2022; Kross et al., 2021; Verduyn et al., 2022). Specifically, contemporary frameworks emphasize that the psychological benefits of digital engagement depend heavily on the presence of real-time reciprocity and contextual warmth (Verduyn et al., 2022).

Live interactive singing represents an ideal case for this more granular analysis. A foundational multi-study investigation found that social media use is rarely perceived as social interaction by users: approximately 75% of users reported no social interaction after 5–10 min of use, and only 2–4% of all reported interactions occurred through social media (Hall, 2018). The behaviors that did qualify shared three features: synchrony, directed exchange, and focused mutual attention—the same features that characterize live interactive singing as defined above. Empirical findings corroborate the unique relational power of this modality: compared to unstructured online conversations, synchronous group singing has been shown to yield significantly greater improvements in both social participation and self-efficacy, highlighting that the embodied co-performance—rather than mere social contact—is the active ingredient for psychological restoration (Schäfer, 2023). From the perspective of self-determination theory, live interactive singing may simultaneously satisfy the fundamental psychological needs for autonomy, competence, and relatedness (Deci et al., 2017; Ryan and Deci, 2000). Specifically, viewers exercise autonomy by choosing to actively co-perform rather than passively watch, build competence through successful vocal coordination, and fulfill relatedness through shared agency and emotional resonance with the streamer and peers (Li and Guo, 2021; Onderdijk et al., 2021).

The need-to-belong hypothesis posits that humans possess an innate drive to form stable interpersonal bonds, and that the frustration of this drive produces loneliness (Baumeister and Leary, 1995). Whether a digital behavior satisfies this need depends on its capacity to generate subjective social inclusion and mutual recognition. General active digital participation is consistently associated with positive psychological outcomes only when engagement involves genuine reciprocal exchange (Godard and Holtzman, 2024; Verduyn et al., 2022), a condition naturally facilitated by the highly synchronous “cyber-social relations” of live streaming (Scheibe et al., 2022). For international students, this proposition acquires additional specificity through the social compensation hypothesis: individuals structurally disadvantaged in offline social environments use online platforms to compensate for social deficits (Bessiere et al., 2008; Tice, 1993; Caplan, 2003; O’Day and Heimberg, 2021). Live interactive singing on native-language platforms may therefore represent a particularly effective form of compensation: it provides culturally congruent emotional expression, requires active co-construction (Lu et al., 2018), and occurs within a community of shared cultural identity (Kianpour et al., 2025). The present study thus examines live interactive singing not as generic “active use” but as a functionally specific digital behavior whose characteristics align with the theoretical criteria for genuine mediated social interaction (Hall, 2018). This study therefore proposes:

*Hypothesis 1*: Live interactive singing behavior in live streaming is negatively associated with loneliness.

### 2.3 Social connection

Building on these theoretical foundations, active digital engagement functions as a critical behavioral catalyst for relational closeness. In live streaming contexts, active participation is consistently linked to elevated social presence and subsequent social connectedness (Chen and Liao, 2022; Goh and Tandoc, 2024). Within this ecosystem, live interactive singing fosters a unique environment where vocal synchrony, shared musical emotion, and real-time audience response converge to produce robust social presence—the perception of connecting with others as “real individuals” within a mediated environment (Biocca et al., 2003). Unlike traditional forms of online communication that rely on static text, the rich verbal and visual cues inherent in live streaming convey interpersonal warmth and emotional states far more effectively, thereby intensifying the experience of “being there” with others (Taieb et al., 2026).

The pathway from this mediated social connection to reduced loneliness is well-established. Increased social engagement on social media is associated with lower loneliness through daily feelings of connectedness, independent of whether users receive direct feedback (Deters and Mehl, 2013). Longitudinal evidence confirms that intimacy and connectedness on social media predict declines in loneliness over time (Sohn et al., 2019). Crucially, directed communication with close ties, as opposed to passive broadcast communication, is the form most consistently linked to enhanced connection and reduced loneliness (Burke and Kraut, 2016; Zhang et al., 2021). Qualitative research adds a vital caveat: social connection must be perceived as genuine; superficial interactions lacking focused, reciprocal exchange fail to be associated with lower isolation (Miguel et al., 2025). This caveat underscores why live interactive singing’s functional characteristics—synchrony, directedness, and emotional depth—are theoretically important: they provide the genuine relational substance necessary to convert behavioral participation into psychological relief. This study therefore proposes:

*Hypothesis 2a*: Social connection mediates the association between live interactive singing and loneliness.

### 2.4 Social support

As the downstream outcome in the sequential process described earlier, perceived social support—the evaluation that emotional, instrumental, and informational resources are available when needed (Wills and Shinar, 2000)—represents a second, conceptually distinct mechanism. Theoretically, this stable cognitive appraisal develops from accumulated moments of situational connection and is associated with lower isolation. Social capital theory provides the foundation: online interactions generate both bonding capital (emotional support from close ties) and bridging capital (resources from weak ties), both of which constitute perceived support that mitigates loneliness (Zhang et al., 2021).

Perceived social support consistently emerges as the outcome most strongly associated with active social media use, with effect sizes substantially larger than associations with any other mental health or wellbeing indicator (Godard and Holtzman, 2024; Meier and Reinecke, 2021). Furthermore, the extended active–passive model specifies that support generation requires reciprocal, emotionally warm exchanges (Verduyn et al., 2022)—precisely the kind of mutual emotional vulnerability that live interactive singing is theoretically positioned to support. Among socially anxious individuals, perceived online social support is uniquely associated with greater wellbeing even when offline support is absent (O’Day and Heimberg, 2021). International students occupy an analogous structural position: language barriers and cultural unfamiliarity often create offline social deficits, rendering home-language online channels the primary conduit through which meaningful support can be perceived and utilized (Jarrar and Nweke, 2025; Tang et al., 2025). This study therefore proposes:

*Hypothesis 2b*: Perceived social support mediates the association between live interactive singing and loneliness.

Although typically treated as parallel mediators (e.g., Zhang et al., 2021), theoretical and empirical reasoning suggests social connection and social support represent sequential stages. Social connection captures the immediate, situational perception of belonging; perceived social support reflects a downstream evaluation that resources are available (Wills and Shinar, 2000). Social presence theory positions others’ perceived “real” presence as the experiential foundation upon which deeper relational constructs—trust, closeness, and eventually perceived support—are progressively built (Biocca et al., 2003). This sequential pathway from presence to connectedness to wellbeing has been confirmed in live-streaming contexts (Chen and Liao, 2022; Goh and Tandoc, 2024).

Social capital theory illuminates this ordering: the immediate perception of co-presence may be associated with relational trust, which over time crystallizes into the perception of reliable social resources (Lin, 1999). Initial online connection has been theorized as a necessary antecedent for richer support perceptions (Zhang et al., 2021). Qualitative evidence reinforces this: digital nomads who converted bridging capital into bonding capital could do so only when initial connection was followed by sustained, relationally meaningful interaction; when encounters remained shallow, they failed to develop into perceived support (Miguel et al., 2025). This study therefore proposes:

*Hypothesis 3*: Social connection and social support sequentially mediate the association between live interactive singing and loneliness.

### 2.5 Fear of missing out

Fear of missing out is a pervasive apprehension that others are having rewarding experiences from which one is absent, accompanied by a compulsive desire to remain continually connected to what others are doing (Przybylski et al., 2013; Tandon et al., 2021). Within self-determination theory, FoMO indexes extrinsically regulated, anxiety-driven engagement rather than autonomously motivated relational interest (Deci et al., 2017; Ryan and Deci, 2000). Conceptualizing this dynamic as a maladaptive coping strategy for frustrated psychological needs (Putri et al., 2026) clarifies why high-FoMO users default to compulsive monitoring rather than engaging in autonomous relational exchanges.

Three converging lines of evidence establish FoMO as a boundary condition on digital engagement outcomes. First, a meta-analysis of over 55,000 participants documents a robust positive association between trait FoMO and problematic internet use, amplified under conditions of stress and uncertainty (Akbari et al., 2021). Second, high FoMO is consistently associated with elevated digital stress and reduced relational satisfaction, mediated through anxiety and compulsive use patterns rather than volume of use alone (Hall et al., 2021; Khetawat and Steele, 2023). Third, qualitative work with mobile populations is consistent with the finding that anxiety-driven pursuit of constant connectivity produces shallow encounters that reinforce rather than alleviate loneliness (Miguel et al., 2025). Applied to live interactive singing, this evidence base implies that FoMO should attenuate the relational yield of participation not by reducing its frequency but by altering its motivational quality: at high FoMO, the same behavior is more likely to be performed in an anxious, monitoring mode that fails to produce social presence, disclosure, and reciprocal recognition.

This motivational-quality logic predicts that FoMO should specifically attenuate the path from live interactive singing to social connection. State social connection is generated in the moment of interaction, through felt mutual recognition and emotional resonance with co-participants (Baumeister and Leary, 1995; Biocca et al., 2003). However, at high FoMO, the participant’s cognitive resources and attention are divided between the present co-singing exchange and an absent comparator—what others elsewhere are doing. Compulsive monitoring and divided attention may undermine the focused mutual attention through which social presence is associated with genuine social connection (Al-Furaih and Al-Awidi, 2021; Rozgonjuk et al., 2019). The behavior is performed, but the typical relational correlate may not follow. This prediction is consistent with experimental and review evidence that anxiety-elevated digital engagement produces shallower, less connection-generating exchanges (Akbari et al., 2021; Putri et al., 2026).

*Hypothesis 4*: Fear of missing out negatively moderates the positive association between live interactive singing and social connection.

FoMO should also attenuate the path from live interactive singing to perceived social support. Perceived support requires that interactional partners be apprehended as warm, reliable, and emotionally available, which in turn requires that the participant attend to the relational quality of the interaction (Wills and Shinar, 2000). At high FoMO, the participant’s anxious vigilance about missed opportunities elsewhere shifts attentional and motivational resources away from the present community (Al-Furaih and Al-Awidi, 2021; Rozgonjuk et al., 2019), making it harder to register the emotional warmth and reliability cues from which support perceptions are constructed. The same singing session that yields a sense of “someone here who understands” under low FoMO yields a more transactional, monitoring-mode encounter under high FoMO.

*Hypothesis 5*: Fear of missing out negatively moderates the positive association between live interactive singing and perceived social support.

Beyond the mediated paths, FoMO should also moderate the direct association between live interactive singing and loneliness. The direct path captures relational benefits not fully accounted for by social connection or perceived social support—plausibly including affective, expressive, and cultural-resonance components of singing participation that operate outside the mediator constructs measured here. These affective benefits depend on autonomous, present-focused engagement (Deci et al., 2017; Ryan and Deci, 2000); when participation is performed under FoMO-driven extrinsic regulation, the same behavior is unlikely to yield the same affective lift. The direct association between live interactive singing and lower loneliness should therefore be weaker at higher levels of FoMO.

*Hypothesis 6*: Fear of missing out moderates the direct negative association between live interactive singing and loneliness, such that this association is weaker at higher levels of fear of missing out.

Finally, because FoMO is hypothesized to attenuate the entry point of the serial pathway (the LISB → social connection link, H4), it should also attenuate the cumulative serial indirect effect that runs through social connection and onward to perceived social support and loneliness. If situational connection is the experiential foundation upon which support perceptions are progressively built (Biocca et al., 2003; Lin, 1999), then a weakened foundation should yield a weakened cumulative pathway. This prediction differs from H4–H6 in its scope: rather than identifying a single moderated link, it specifies that the entire serial mechanism through which live interactive singing is associated with lower loneliness operates more weakly at high FoMO.

*Hypothesis 7*: Fear of missing out moderates the serial indirect association of live interactive singing with loneliness through social connection and perceived social support.

**Figure 1:**
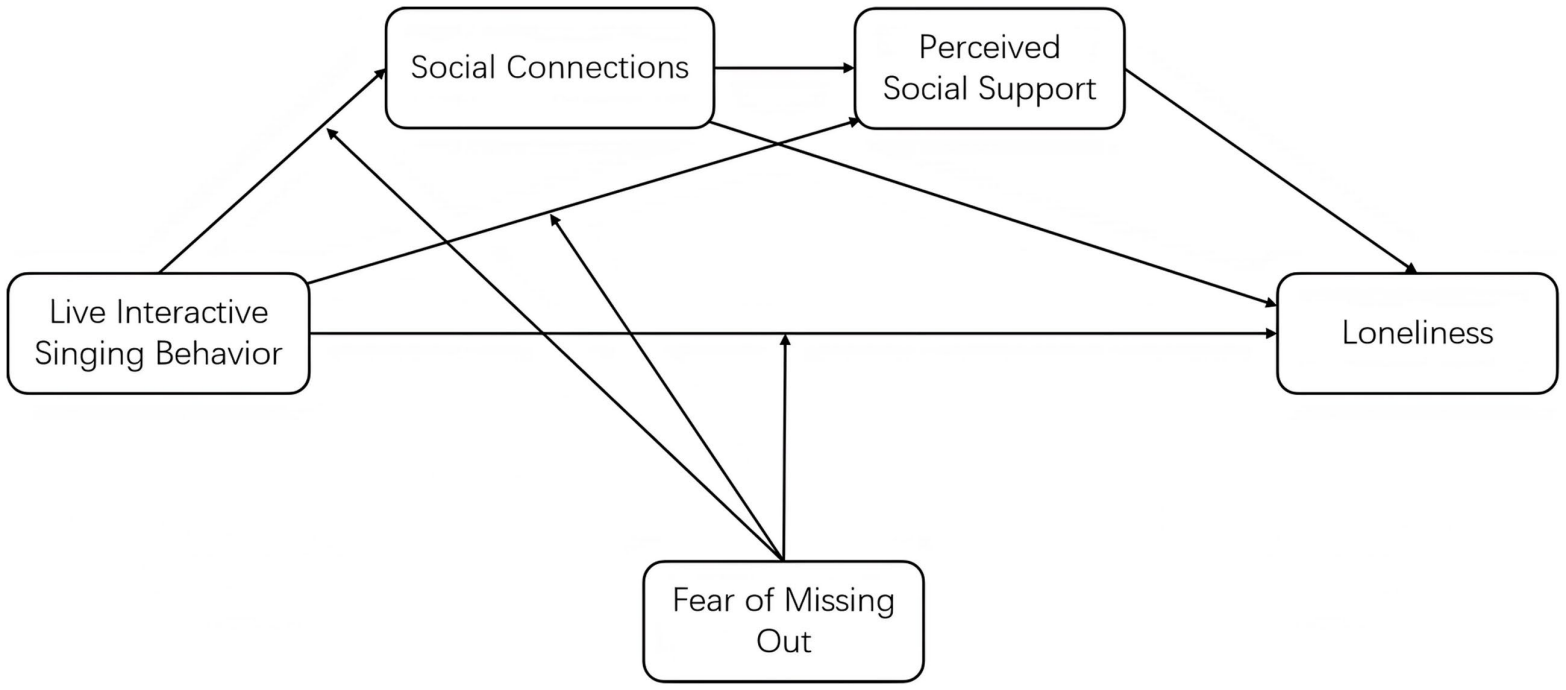
Conceptual model.

Figure 1 presents the conceptual model of this study.

## 3 Methods

### 3.1 Participants and procedure

The present study employed a cross-sectional online survey design targeting international students who live abroad and actively use native-language live-streaming platforms. Data collection was contracted to a Chinese commercial market research firm (Chongqing Rounen Film and Television Media Co., Ltd., Chongqing, China). The firm administered the survey through their online platform between February, 2026 and March, 2026. Eligibility was verified by the firm using a three-stage screening procedure (current overseas residence; using the native language platform every week for the past month; at least four live interactive singing sessions in the past month). Recruitment relied on the market research firm’s pre-existing panel of internationally-mobile Chinese respondents, supplemented by referral recruitment within the panel (panel members were invited to share the survey link with eligible peers in their host-country networks). This combined panel-based and snowball strategy was chosen to maximize reach across geographically dispersed host countries while enforcing strict eligibility screening. The implications of this non-probability sampling approach for generalizability are addressed in the Limitations. All participants provided informed consent prior to participation and were informed that responses would remain anonymous and be used exclusively for academic research. No personally identifiable information was collected. This study has received ethical approval prior to data collection from the Ethical Review Committee of School of Journalism and Communication (CQUSJC2026012), Chongqing University, China.

To ensure data quality, the survey embedded three attention-check items. Responses failing these checks, exhibiting patterned responding, or completed in under 3 min were excluded. The survey was administered in Simplified Chinese, the participants’ native language. All instruments originally published in English underwent forward–backward translation by two independent bilingual researchers (Chinese L1, English L2 doctoral level). Discrepancies were resolved through consensus, with reference to a previously validated Chinese-language version of each scale.

After data screening, the final valid sample consisted of *N* = 685 participants. All respondents were citizens of Mainland China currently studying abroad, in line with the eligibility criterion of native-language live-streaming platform use. The sample was balanced by gender (345 male, 50.4%; 340 female, 49.6%) and by academic level (338 undergraduates, 49.3%; 347 graduate students, 50.7%). Host countries reflected the principal destinations of Mainland Chinese international students worldwide: the United States (*n* = 153, 22.3%), the United Kingdom (*n* = 127, 18.5%), Canada (*n* = 85, 12.4%), Australia (*n* = 80, 11.7%), Japan (*n* = 59, 8.6%), Germany (*n* = 50, 7.3%), France (*n* = 39, 5.7%), the Netherlands (*n* = 29, 4.2%), South Korea (*n* = 25, 3.6%), New Zealand (*n* = 21, 3.1%), and other destinations (*n* = 17, 2.5%). Mean age was 23.84 years (SD = 3.51, range 18–34), with the expected difference between undergraduates (M = 21.19, SD = 1.74) and graduates (M = 26.41, SD = 2.81). Length of time in the host country averaged 20.24 months (SD = 14.23, median = 18, range 1–84). Self-reported quality of offline social life in the host country was measured on a 5-point scale (M = 3.09, SD = 1.01).

### 3.2 Measures

All focal constructs were assessed with established psychological measures. Where adaptation was required to fit the live-streaming co-singing context, three principles were applied. First, where the construct was conceptualized as a stable trait or general state (loneliness, fear of missing out), original wording was retained verbatim. Second, where the construct was conceptualized as a state-level response to a specific interactional context (social connection, perceived online social support), the instructional stem was modified to direct participants’ attention to their experience during live interactive singing while item content was preserved; one social-connection item also required a single-phrase substitution (detailed below). Third, where no item-level scale existed for the focal behavior (live interactive singing), items were adapted from two validated source instruments (Taieb et al., 2026; Ozimek et al., 2023) as documented in the LISB section. Adaptations were reviewed by two bilingual researchers familiar with the source literatures. Verification of the resulting measurement model—confirmatory factor analysis, composite reliability, convergent and discriminant validity, and common method bias diagnostics—is reported in Section 4.1. Composite scores were computed by averaging item responses after reverse-coding where appropriate.

Live interactive singing behavior was assessed with a 5-item adapted scale. Three items were adapted from the Streamer–Viewer Interaction (SVI) dimension of the Online Live Streaming Interaction (OLSI) scale developed and cross-culturally validated by Taieb et al. (2026). Two items were adapted from Ozimek et al.'s (2023) Social Media Activity Questionnaire (SMAQ). Live interactive singing behavior (*α* = 0.82, M = 3.18, SD = 0.64, KMO = 0.85, variance explained = 57.79%) was defined as the extent to which a viewer actively and reciprocally participates in singing with a streamer—and with co-present peers—during a live broadcast through real-time vocal engagement. Items were recontextualized to the embodied vocal modality of live interactive singing while preserving the synchrony and directedness emphasized in both source instruments. Following the SMAQ format, participants reported how often they engaged in each behavior on a 5-point frequency scale (1 = never, 5 = very often). Example items included: “I sing together with the streamer during live streams” (M = 3.14, SD = 0.86; adapted from Ozimek et al., 2023), “I join in singing when the streamer invites viewers to participate” (M = 3.19, SD = 0.83; adapted from Ozimek et al., 2023), “During live singing, I respond to the streamer in real time through singing or vocal participation” (M = 3.18, SD = 0.86; adapted from Taieb et al., 2026), “During live singing, the streamer responds to or acknowledges my singing participation” (M = 3.18, SD = 0.81; adapted from Taieb et al., 2026), and “During live singing, I interact through singing or vocal participation with others who are taking part at the same time” (M = 3.20, SD = 0.87; adapted from Taieb et al., 2026).

Social connection (*α* = 0.92, M = 4.40, SD = 0.87, KMO = 0.96, variance explained = 56.88%) served as the first mediator. It was measured with the 10-item University of British Columbia State Social Connection Scale (Lok and Dunn, 2023), which assesses momentary feelings of connection following a specific social interaction. Participants were instructed to recall their feelings during a co-singing session. Two adaptations were made. The instructional stem was contextualized to direct participants to their experience during the co-singing session, consistent with the scale’s design as a state-level measure following a specific interaction (Lok and Dunn, 2023). One item (SC05) was modified by substituting “the live-stream environment around me” for “the world around me,” because the original referent is ambiguous in a digitally mediated context. The remaining nine items were administered verbatim. Four items were reverse-coded, with higher scores indicating greater social connection. Responses were made on a 7-point Likert scale (1 = strongly disagree, 7 = strongly agree). Example items included: “I felt distant from people” (reverse-coded; M = 4.43, SD = 1.13), “I felt like I was able to connect with other people” (M = 4.41, SD = 1.16), “I felt disconnected from the live-stream environment around me” (reverse-coded; M = 4.39, SD = 1.16), and “I had a sense of belonging” (M = 4.41, SD = 1.16).

Perceived social support (*α* = 0.83, M = 3.28, SD = 0.55, KMO = 0.89, variance explained = 54.72%) served as the second mediator. It was assessed with a 6-item adaptation of the Online Social Support Scale (Nick et al., 2018). Items were contextualized to refer specifically to the live-stream co-singing community (e.g., “Someone in the co-singing community to turn to.” rather than the source scale’s generic “someone online”). This contextualization was necessary because the construct under study is support perceived from a specific interactional partner set, not online support in general; without it, participants might have reported on unrelated online networks. Item content otherwise followed Nick et al. (2018). This measure was selected over general support scales because the study focused specifically on support perceived through online interaction. Participants rated support availability on a 5-point scale (1 = none of the time, 5 = all of the time). Example items included: “Someone in the co-singing community to turn to for suggestions about how to deal with a personal problem” (M = 3.26, SD = 0.74), “Someone in the co-singing community who understands your problems” (M = 3.27, SD = 0.72), and “Someone in the co-singing community who shows you love and affection” (M = 3.29, SD = 0.76).

Loneliness (*α* = 0.89, M = 2.49, SD = 0.52, KMO = 0.94, variance explained = 56.48%) served as the outcome. It was measured with the 8-item short form of the UCLA Loneliness Scale, Version 3 (Hays and DiMatteo, 1987; Russell, 1996). Because loneliness was conceptualized as a generalized psychological state, the items were administered verbatim without contextual modification. Two items were reverse-coded so that higher scores indicated greater loneliness. Participants rated how often each statement described them on a 4-point scale (1 = never, 4 = always). Example items included: “I lack companionship” (M = 2.49, SD = 0.70), “There is no one I can turn to” (M = 2.49, SD = 0.70), “I feel isolated from others” (M = 2.48, SD = 0.72), and “People are around me but not with me” (M = 2.48, SD = 0.69).

FoMO (α = 0.90, M = 3.10, SD = 0.62, KMO = 0.95, variance explained = 53.58%) served as the moderator. It was measured with the 10-item Fear of Missing Out Scale (Przybylski et al., 2013). FoMO was treated as a relatively stable dispositional tendency; therefore, the original item wording was retained without contextual modification. Participants rated each statement on a 5-point scale (1 = not at all true of me, 5 = extremely true of me). Example items included: “I fear others have more rewarding experiences than me” (M = 3.12, SD = 0.88), “I get anxious when I do not know what my friends are up to” (M = 3.14, SD = 0.85), and “It bothers me when I miss an opportunity to meet up with friends” (M = 3.09, SD = 0.85).

Three covariates were included in all models: gender, academic level (1 = undergraduate, 2 = graduate), and self-reported quality of offline social life in the host country (5-point scale). Offline social-life quality was included to test whether the associations involving Live interactive singing behavior were evident above and beyond participants’ baseline offline social resources.

### 3.3 Data analysis

Several preliminary analyses were conducted before testing the hypothesized model. Descriptive statistics, Pearson correlations, and internal-consistency coefficients (Cronbach’s *α*) were computed for all focal variables. Distributional assumptions were examined using skewness and kurtosis; values of |skewness| < 2 and |kurtosis| < 7 were treated as acceptable. Common method bias was assessed with Harman’s single-factor test by entering all focal items into an unrotated principal-components analysis. A first factor accounting for less than 40% of the total variance was taken as evidence that common method variance was unlikely to dominate the data. Multicollinearity was evaluated using variance inflation factors, with VIFs below 5 indicating acceptable levels.

The hypothesized moderated serial mediation model was tested with PROCESS for SPSS (Hayes et al., 2022), using Model 85. Live interactive singing behavior was specified as the predictor, social connection as the first mediator, perceived social support as the second mediator, loneliness as the outcome, and FoMO as the moderator. Gender, academic level, and offline social-life quality were included as covariates in all equations. Live interactive singing behavior and FoMO were mean-centered before analysis. HC3 heteroskedasticity-consistent standard errors were used throughout. Indirect effects were evaluated using 5,000 bootstrap resamples and bias-corrected 95% confidence intervals (CI); effects were considered significant when the interval did not include zero. Conditional effects were probed at low (−1 SD), mean, and high (+1 SD) levels of FoMO. Johnson-Neyman regions of significance were also estimated to identify the moderator values at which conditional effects became significant or nonsignificant.

As a supplementary robustness check, the PROCESS Model 85 analysis was re-estimated separately for undergraduates (*n* = 338) and graduates (*n* = 347). In these subgroup models, gender and offline social-life quality were retained as covariates, whereas academic level was omitted because it was constant within subgroup. This analysis examined whether the overall pattern of conditional indirect effects was comparable across academic groups.

## 4 Results

### 4.1 Measurement model validation

Prior to hypothesis testing, the measurement properties of all five focal constructs—live interactive singing behavior (LISB), social connection, perceived social support, fear of missing out, and loneliness—were evaluated through confirmatory factor analysis (CFA), reliability estimation, and convergent and discriminant validity assessment. The CFA was estimated using maximum likelihood estimation in semopy. Model fit was evaluated against the conventional criteria of CFI ≥ 0.95, TLI ≥ 0.95, and RMSEA ≤ 0.06. Convergent validity was assessed via standardized factor loadings (≥ 0.50), composite reliability (CR ≥ 0.70), and average variance extracted (AVE ≥ 0.50). Discriminant validity was evaluated using both the Fornell–Larcker criterion and the heterotrait–monotrait (HTMT) ratio, with HTMT values < 0.85 considered evidence of discriminant validity.

#### 4.1.1 Confirmatory factor analysis

A five-factor measurement model with 39 indicators loading on their respective latent constructs was estimated. The model demonstrated excellent fit to the data: *χ*^2^(692) = 769.82, *p* = 0.021, CFI = 0.993, TLI = 0.993, RMSEA = 0.013, GFI = 0.937, AGFI = 0.933. The chi-square test was statistically significant, but this is expected given the test’s known sensitivity to sample size at *N* = 685; the relative chi-square (*χ*^2^/df = 1.11) was well below the conservative threshold of 3.0. All 39 standardized factor loadings were statistically significant (*p* < 0.001) and ranged from 0.65 to 0.78, exceeding the 0.50 threshold for adequate item reliability. As a supplementary single-construct analysis, a CFA on the five LISB items alone produced excellent fit (*χ*^2^(5) = 2.62, *p* = 0.758; CFI = 1.00; TLI = 1.00; RMSEA = 0.000), with all five items loading between 0.66 and 0.73 on the latent LISB factor. These results support the unidimensional structure of the adapted LISB scale and the overall measurement model. Standardized loadings for all items are reported in Table 1.

**Table 1:** Standardized factor loadings, item means, and standard deviations.

Item	*λ* (std)	M	SD
LISB1	0.66	3.14	0.86
LISB2	0.66	3.19	0.83
LISB3	0.72	3.18	0.86
LISB4	0.66	3.18	0.81
LISB5	0.73	3.20	0.87
SC1 (R)	0.77	4.43	1.13
SC2 (R)	0.71	4.39	1.16
SC3 (R)	0.69	4.40	1.14
SC4	0.73	4.41	1.16
SC5 (R)	0.71	4.39	1.16
SC6	0.73	4.42	1.13
SC7	0.69	4.40	1.14
SC8	0.74	4.40	1.15
SC9	0.70	4.41	1.16
SC10	0.74	4.40	1.13
SS1	0.70	3.26	0.74
SS2	0.69	3.27	0.72
SS3	0.70	3.28	0.74
SS4	0.65	3.30	0.74
SS5	0.66	3.28	0.73
SS6	0.66	3.29	0.76
FM1	0.74	3.12	0.88
FM2	0.68	3.10	0.84
FM3	0.69	3.09	0.83
FM4	0.69	3.14	0.85
FM5	0.67	3.10	0.84
FM6	0.71	3.10	0.86
FM7	0.70	3.09	0.85
FM8	0.70	3.11	0.84
FM9	0.68	3.10	0.84
FM10	0.68	3.10	0.84
LO1	0.70	2.49	0.70
LO2	0.71	2.49	0.70
LO3 (R)	0.70	2.49	0.70
LO4	0.75	2.50	0.71
LO5	0.73	2.48	0.72
LO6 (R)	0.66	2.49	0.70
LO7	0.71	2.49	0.71
LO8	0.71	2.48	0.69

(R), reverse-coded item; λ, standardized factor loading. All loadings significant at *p* < 0.001.

#### 4.1.2 Reliability and convergent validity

Internal consistency was high for all five constructs, with Cronbach’s α ranging from 0.82 (LISB) to 0.92 (social connection). Composite reliability values ranged from 0.82 to 0.92, all exceeding the 0.70 benchmark. Average variance extracted (AVE) ranged from 0.46 to 0.52, with values for social connection (0.52) and loneliness (0.50) meeting the conventional 0.50 threshold and values for LISB (0.47), perceived social support (0.46), and fear of missing out (0.48) falling marginally below it. AVE below 0.50 is acceptable when CR exceeds 0.60, on the grounds that convergent validity is supported when reliability evidence is strong. Given that all CR values in the present study substantially exceeded this benchmark and that all standardized loadings exceeded 0.65, convergent validity was considered adequate (see Table 2).

**Table 2:** Reliability and convergent validity indices for the five latent constructs.

Construct	*k*	*α*	CR	AVE	*λ* range
Live interactive singing behavior	5	0.82	0.82	0.47	0.66–0.73
Social connection	10	0.92	0.92	0.52	0.69–0.78
Perceived social support	6	0.83	0.84	0.46	0.65–0.70
Fear of missing out	10	0.90	0.90	0.48	0.67–0.74
Loneliness	8	0.89	0.89	0.50	0.66–0.75

*k*, number of items; *α*, Cronbach’s alpha; CR, composite reliability; AVE, average variance extracted; *λ*, standardized factor loading.

#### 4.1.3 Discriminant validity

Discriminant validity was assessed using two complementary criteria. First, applying the Fornell–Larcker criterion, the square root of each construct’s AVE was compared with its correlations with all other latent constructs (Table 3). For every construct, the square root of AVE exceeded the absolute value of its correlations with all other constructs in the model. The smallest √AVE value (0.68, for perceived social support) exceeded the largest inter-construct correlation, which was the negative correlation between perceived social support and loneliness (*r* = −0.53). Second, heterotrait–monotrait (HTMT) ratios were computed (Table 4). All HTMT values fell substantially below the conservative 0.85 threshold, with the largest value being 0.53 (perceived social support ↔ loneliness). HTMT values involving fear of missing out were notably small (all ≤ 0.24), reflecting its conceptual distinctness as a dispositional moderator rather than a relational construct. Together, the Fornell–Larcker and HTMT analyses provide convergent evidence that the five constructs are empirically distinct.

**Table 3:** Fornell–Larcker criterion: latent correlations and √AVE.

Construct	1	2	3	4	5
1. Live interactive singing behavior	**0.69**				
2. Social connection	0.38	**0.72**			
3. Perceived social support	0.28	0.51	**0.68**		
4. Fear of missing out	−0.06	0.05	−0.06	**0.70**	
5. Loneliness	−0.31	−0.47	−0.53	0.24	**0.71**

Bold values on the diagonal are square roots of AVE; off-diagonal values are inter-construct correlations from the CFA. Discriminant validity is supported when each diagonal value exceeds all off-diagonal values in its row and column.

**Table 4:** Heterotrait–monotrait (HTMT) ratios.

Construct	1	2	3	4	5
1. Live interactive singing behavior	—				
2. Social connection	0.38	—			
3. Perceived social support	0.28	0.51	—		
4. Fear of missing out	0.07	0.06	0.07	—	
5. Loneliness	0.31	0.47	0.53	0.24	—

All HTMT values fall below the 0.85 threshold, supporting discriminant validity.

#### 4.1.4 Common method bias

Because all measures relied on participant self-report, common method bias was assessed using two diagnostics. First, Harman’s single-factor test was conducted by entering all 39 indicators into an unrotated principal-components analysis. The first unrotated factor accounted for 28.54% of the total variance, well below both the conservative 40% threshold and the more commonly cited 50% threshold. Second, the excellent fit of the five-factor measurement model reported in 4.1.1—and the substantially poorer fit that would obtain if a single common method factor accounted for the data—provides additional evidence against pervasive method bias. Although Harman’s test has limited diagnostic sensitivity, the convergence of the two diagnostics suggests that common method bias is unlikely to substantially distort the substantive findings.

### 4.2 Hypothesis testing

Hypothesis 1 predicted that live interactive singing behavior would be negatively associated with loneliness among international students. As shown in Table 5, this hypothesis was supported. The direct association between live interactive singing behavior and loneliness was negative and statistically significant (*b* = −0.10, *p* < 0.001).

**Table 5:** Mediation model.

Path	*b*	95% CI	*p*
Live interactive singing behavior → social connection	0.45	[0.360, 0.547]	<0.001
Social connection → perceived social support	0.24	[0.188, 0.284]	<0.001
Live interactive singing behavior → perceived social support	0.09	[0.029, 0.153]	0.004
Perceived social support → loneliness	−0.24	[−0.313, −0.177]	<0.001
Social connection → loneliness	−0.14	[−0.182, −0.098]	<0.001
Live interactive singing behavior → loneliness (direct association)	−0.10	[−0.150, −0.046]	<0.001
Live interactive singing behavior → social connection → loneliness	−0.06	[−0.087, −0.042]	/
Live interactive singing behavior → perceived social support → loneliness	−0.02	[−0.040, −0.007]	/
Live interactive singing behavior → social connection → perceived social support → loneliness	−0.03	[−0.038, −0.017]	/

Hypothesis 2a predicted that social connection would mediate the association between live interactive singing behavior and loneliness. This hypothesis was supported. Live interactive singing behavior was positively associated with social connection (*b* = 0.45, *p* < 0.001), and social connection was negatively associated with loneliness (*b* = −0.14, *p* < 0.001). The indirect association through social connection was statistically significant (*b* = −0.06, 95% CI [−0.087, −0.042]).

Hypothesis 2b predicted that perceived social support would mediate the association between live interactive singing behavior and loneliness. This hypothesis was also supported. Live interactive singing behavior was positively associated with perceived social support (*b* = 0.09, *p* = 0.004), and perceived social support was negatively associated with loneliness (*b* = −0.24, *p* < 0.001). The indirect association through perceived social support was statistically significant (*b* = −0.02, 95% CI [−0.040, −0.007]).

Hypothesis 3 predicted that social connection and perceived social support would sequentially mediate the association between live interactive singing behavior and loneliness. This hypothesis was supported. The serial indirect association was statistically significant (*b* = −0.03, 95% CI [−0.038, −0.017]).

**Figure 2:**
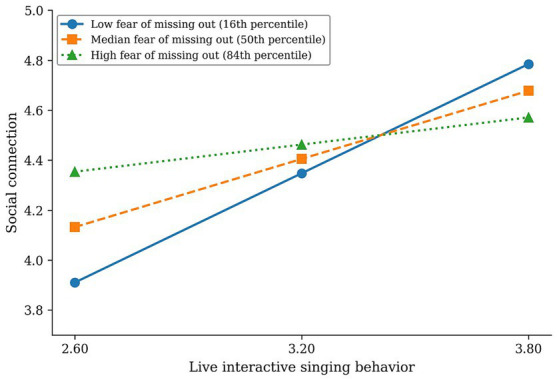
Simple slopes for social connection.

Hypothesis 4 predicted that fear of missing out would negatively moderate the positive association between live interactive singing behavior and social connection. This hypothesis was supported. The interaction between live interactive singing behavior and fear of missing out was statistically significant (*b* = −0.46, *p* < 0.001). The conditional association was strongest at low fear of missing out (*b* = 0.73), weaker at medium fear of missing out (*b* = 0.46), and weakest at high fear of missing out (*b* = 0.18), although it remained statistically significant at all three levels. Johnson-Neyman analysis indicated that this association became nonsignificant at a fear of missing out value 0.68 standard deviations above the mean; 84.7 percent of the sample fell below this threshold. Figure 2 displays this interaction.

**Figure 3:**
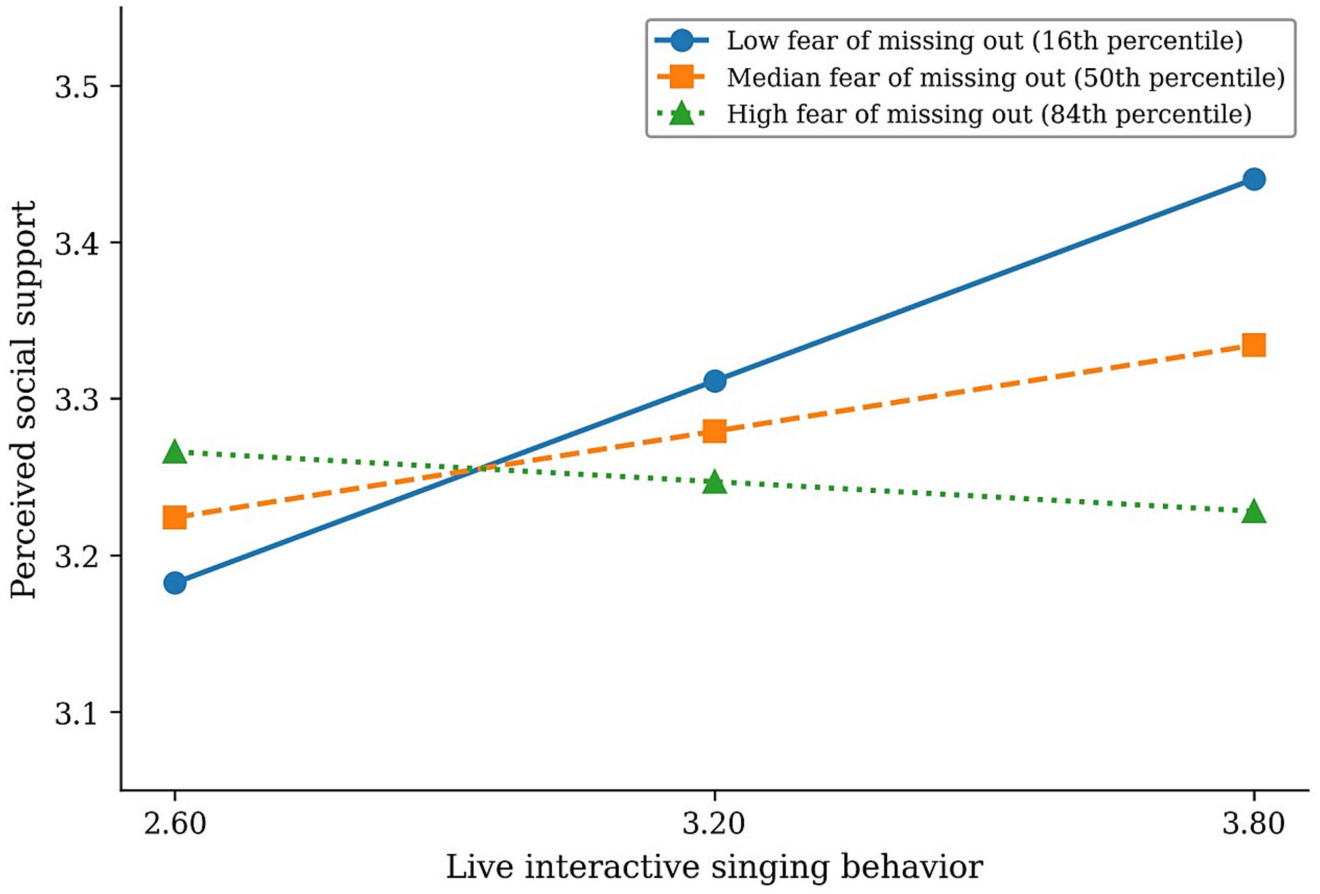
Simple slopes for social support.

Hypothesis 5 predicted that fear of missing out would negatively moderate the positive association between live interactive singing behavior and perceived social support. This hypothesis was supported. The interaction was statistically significant (*b* = −0.21, *p* < 0.001). The conditional association was positive and significant at low and medium fear of missing out, but was not significant at high fear of missing out. Johnson-Neyman analysis indicated that this association became nonsignificant at a fear of missing out value 0.15 standard deviations above the mean; 57.8 percent of the sample fell below this threshold. Figure 3 displays this interaction.

**Figure 4:**
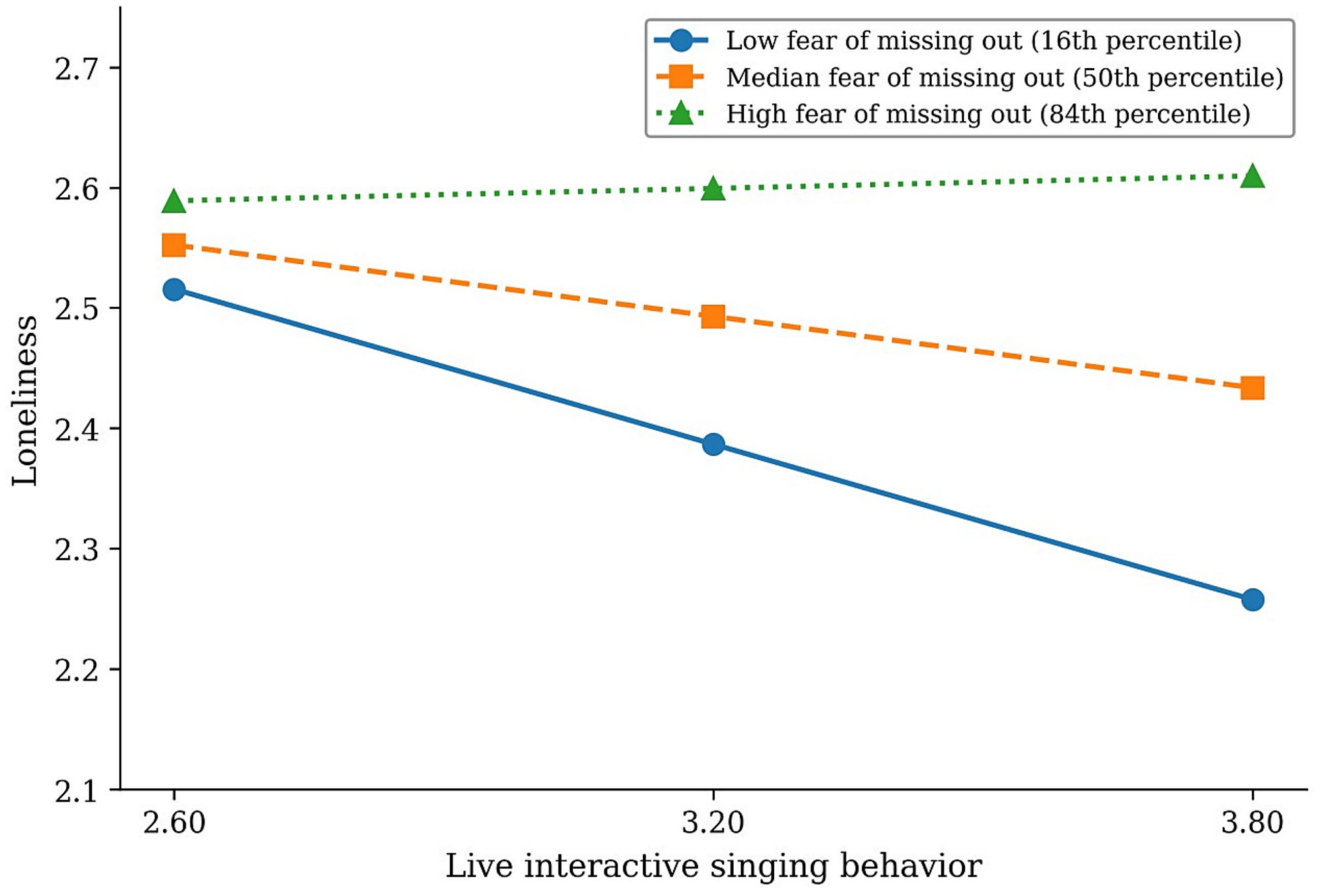
Simple slopes for loneliness.

Hypothesis 6 predicted that fear of missing out would moderate the direct negative association between live interactive singing behavior and loneliness, such that this association would be weaker at higher levels of fear of missing out. This hypothesis was supported. The interaction was statistically significant (*b* = 0.19, *p* < 0.001). The direct association was negative and significant at low fear of missing out (*b* = −0.22, *p* < 0.001) and at medium fear of missing out (*b* = −0.10, *p* < 0.001), but not at high fear of missing out (*b* = 0.02, *p* = 0.612). Johnson-Neyman analysis indicated that the direct association became nonsignificant at a fear of missing out value 0.23 standard deviations above the mean; 65.1 percent of the sample fell below this threshold. Figure 4 presents this interaction.

As shown in Table 6, the index of moderated mediation was significant for the indirect pathway through social connection (index = 0.064, 95% CI [0.038, 0.092]). The indirect association decreased in magnitude from −0.10 at low fear of missing out to −0.03 at high fear of missing out, but remained statistically significant across all three levels. The index of moderated mediation was also significant for the indirect pathway through perceived social support (index = 0.050, 95% CI [0.027, 0.077]). This indirect association was statistically significant at low and medium fear of missing out, but not at high fear of missing out.

**Table 6:** Moderated mediation model.

Path	*b*	95% CI	*p*
Interaction effects			
Live interactive singing behavior * fear of missing out → social connection	−0.46	[−0.599, −0.314]	<0.001
Live interactive singing behavior * fear of missing out → perceived social support	−0.21	[−0.290, −0.122]	<0.001
Live interactive singing behavior * fear of missing out → loneliness	0.19	[0.119, 0.269]	<0.001
Conditional effects on social connection			
Fear of missing out (low)	0.73	[0.607, 0.851]	/
Fear of missing out (medium)	0.46	[0.362, 0.549]	/
High fear of missing out	0.18	[0.049, 0.313]	/
Conditional effects on perceived social support			
Fear of missing out (low)	0.22	[0.126, 0.304]	/
Fear of missing out (medium)	0.09	[0.030, 0.154]	/
Fear of missing out (high)	−0.03	[−0.101, 0.038]	/
Conditional direct effects on loneliness			
Fear of missing out (low)	−0.22	[−0.286, −0.144]	/
Fear of missing out (medium)	−0.10	[−0.151, −0.047]	/
Fear of missing out (high)	0.02	[−0.050, 0.084]	/
Conditional indirect effects through social connection			
Fear of missing out (low)	−0.10	[−0.137, −0.068]	/
Fear of missing out (medium)	−0.06	[−0.087, −0.042]	/
Fear of missing out (high)	−0.03	[−0.046, −0.008]	/
Index of moderated mediation	0.06	[0.038, 0.092]	/
Conditional indirect effects through perceived social support			
Fear of missing out (low)	−0.05	[−0.082, −0.028]	/
Fear of missing out (medium)	−0.02	[−0.040, −0.007]	/
Fear of missing out (high)	0.01	[−0.009, 0.026]	/
Index of moderated mediation	0.05	[0.027, 0.077]	/
Conditional indirect effects through the serial pathway			
Fear of missing out (low)	−0.04	[−0.060, −0.027]	/
Fear of missing out (medium)	−0.03	[−0.038, −0.017]	/
Fear of missing out (high)	−0.01	[−0.020, −0.003]	/
Index of moderated mediation	0.03	[0.015, 0.040]	/

Hypothesis 7 predicted that fear of missing out would moderate the serial indirect association between live interactive singing behavior and loneliness through social connection and perceived social support. This hypothesis was supported. The index of moderated mediation for the serial pathway was significant (index = 0.03, 95% CI [0.015, 0.040]). The serial indirect association was statistically significant at low, medium, and high levels of fear of missing out, although its magnitude decreased as fear of missing out increased.

To complement the hypothesis tests with information about the practical magnitude of the associations, the proportion of variance explained was examined for each equation in the moderated serial mediation model. The model accounted for 16.5% of the variance in social connection, *F*(6, 678) = 22.29, *p* < 0.001, adjusted *R*^2^ = 0.157; 24.2% of the variance in perceived social support, *F*(7, 677) = 30.92, *p* < 0.001, adjusted *R*^2^ = 0.234; and 37.8% of the variance in loneliness, *F*(8, 676) = 51.33, *p* < 0.001, adjusted *R*^2^ = 0.371. The incremental variance attributable to the live interactive singing behavior × fear of missing out interaction term was Δ*R*^2^ = 0.050 in the social connection equation (*f*^2^ = 0.059), Δ*R*^2^ = 0.024 in the perceived social support equation (*f*^2^ = 0.032), and Δ*R*^2^ = 0.023 in the loneliness equation (*f*^2^ = 0.037). These interaction effect sizes fall in the small-to-medium range. The direct, indirect, and serial associations are statistically robust at the present sample size but should be understood as modest in absolute magnitude—live interactive singing behavior is one contributing relational factor among many, not a singular determinant of loneliness in this population.

**Figure 5:**
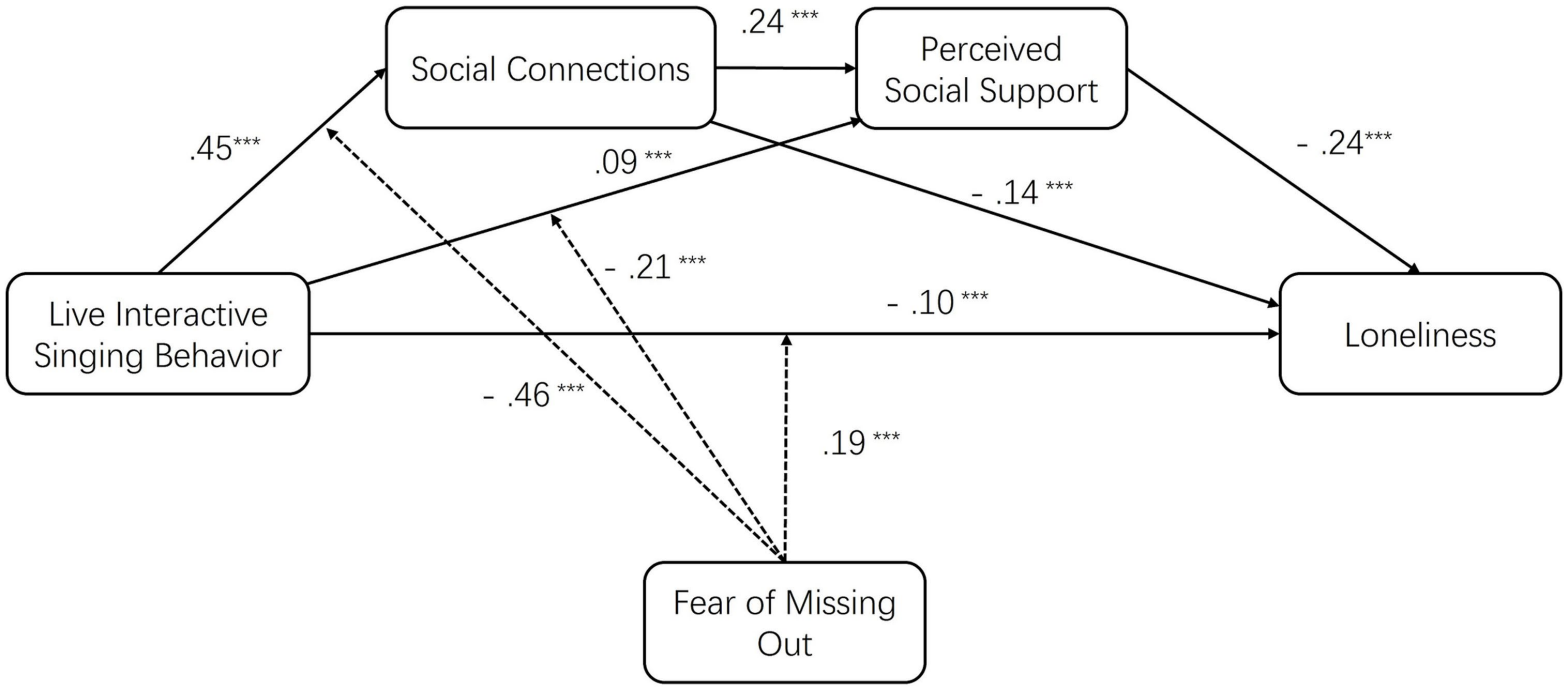
Path coefficients. Solid arrows represent direct path coefficients (b). Dashed arrows from Fear of Missing Out indicate moderation effects; coefficients on the dashed arrows represent the LISB × FoMO interaction term (not a direct effect of FoMO). Coefficients are unstandardized. ^***^p < 0.001.

Figure 5 shows the path coefficients from this study.

### 4.3 Sensitivity analysis

To assess the robustness of the full-sample findings, the analysis was re-estimated separately for undergraduate students (*n* = 338) and graduate students (*n* = 347). Gender and offline social-life quality were retained as covariates, whereas academic level was excluded because it was constant within subgroup.

Across both subgroups, the interaction between live interactive singing behavior and fear of missing out was statistically significant in all model equations, and the indices of moderated mediation were significant for all three indirect pathways. The overall pattern was similar to that observed in the full sample: higher fear of missing out was associated with weaker indirect associations between live interactive singing behavior and loneliness. In the undergraduate subgroup, all three indirect associations were nonsignificant at high fear of missing out. In the graduate subgroup, the pathway through social connection and the serial pathway remained significant at high fear of missing out, whereas the pathway through perceived social support did not. The conditional direct association between live interactive singing behavior and loneliness at the medium level of fear of missing out was significant in the undergraduate subgroup (*b* = −0.13, *p* = 0.001), but did not reach conventional significance in the graduate subgroup (*b* = −0.07, *p* = 0.074). These differences were small in magnitude and may reflect reduced statistical power in the smaller subsamples. No qualitative reversals of the full-sample pattern were observed.

## 5 Discussion

This study examined the associations among live interactive singing behavior, social connection, perceived social support, fear of missing out, and loneliness in a cross-sectional sample of 685 international students.

### 5.1 Summary of findings

First, live interactive singing behavior was negatively associated with loneliness, and this association was consistent with indirect pathways through social connection and perceived social support, both independently and in sequence. In the serial pathway, live interactive singing was positively associated with social connection, social connection was positively associated with perceived social support, and perceived social support was in turn negatively associated with loneliness. This sequential pattern is compatible with a theoretically specified ordering in which situational feelings of belonging precede, and develop into, more stable perceptions of available support.

Second, fear of missing out functioned as a boundary condition that attenuated multiple associations in the model. At elevated levels of fear of missing out, the direct association between live interactive singing behavior and loneliness was no longer significant, and the indirect pathway through perceived social support alone was similarly nonsignificant. The serial mediation pathway remained significant at high levels of fear of missing out, but its magnitude was weakened. These findings suggest that the dispositional orientation accompanying digital engagement may be as relevant to the observed associations as the behavior itself.

Third, the sensitivity analysis indicated that the overall pattern of moderated mediation was broadly consistent across undergraduate and graduate subgroups, with minor differences that were plausibly attributable to reduced statistical power in the smaller subsamples.

### 5.2 Theoretical discussion

The finding that live interactive singing behavior was negatively associated with loneliness among international students supports the social compensation hypothesis. As posited earlier, individuals who face structural constraints in offline social environments often utilize online platforms to address unmet belonging needs (O’Day and Heimberg, 2021; Tang et al., 2025). However, the present findings add critical specificity to this proposition. Given that most effect sizes for general active social media use remain negligible and heterogeneous (Godard and Holtzman, 2024; Kross et al., 2021), identifying which specific behaviors yield genuine relational benefits is essential.

Revisiting the theoretical criteria established by Hall (2018), meaningful mediated social interaction requires synchronous, directed exchanges rather than generic platform use. Live interactive singing—which involves real-time vocal coordination, focused mutual attention, and emotional expression through embodied vocal performance—aligns with these features (Schäfer, 2023). The present finding that this behavior was associated with higher social connection and lower loneliness empirically validates this characterization. Crucially, this association parallels Godard and Holtzman's (2024) meta-analytic observation that perceived social support stands out as a notable exception to the otherwise negligible associations between active use and wellbeing. Live interactive singing may thus represent one of the functionally specific behaviors that accounts for this exception, though this possibility requires direct comparative testing in future research.

Beyond identifying specific behaviors, the serial mediation finding—in which social connection and perceived social support were ordered as sequential links—extends prior work that has typically treated these constructs as parallel, independent mediators. For instance, Zhang et al. (2021) found that perceived social support and social contact independently mediated the association between social media communication and loneliness among older adults. While parallel mediation may suitably describe platforms anchored in pre-existing offline networks (such as Facebook), chat-based live streaming often involves interactions among relative strangers. In such highly transient digital environments, we argue that a sequential specification is theoretically superior: situational social connection may function as a prerequisite bridge. The present study provides evidence that the immediate feeling of being connected during a broadcast may, over time, accumulate into a stable cognitive appraisal of available social support, which is in turn associated with lower loneliness.

This ordering aligns with both social presence theory, which positions the immediate perception of others as the experiential foundation for deeper relational constructs (Biocca et al., 2003; Taieb et al., 2026), and social capital theory, which holds that relational trust must accumulate before translating into reliable resources (Lin, 1999). For international students geographically separated from established networks, this sequential progression highlights a vital pathway for psychological restoration. By co-performing in native-language streams, these students engage in “digital togetherness,” cultivating a diasporic identity that compensates for offline isolation (Kianpour et al., 2025).

However, the social compensation mechanism itself warrants critical reflection. While live interactive singing was associated with lower loneliness in the present sample, presumably through access to a culturally congruent, supportive community, it concurrently risks confining international students within an “international bubble” or a “parallel society” (Ivanova et al., 2025). As noted in recent qualitative inquiries, over-reliance on home-culture digital environments might satisfy immediate belongingness needs but inadvertently distance students from host-national integration and local bridging social capital (Ivanova et al., 2025). Thus, the current findings demarcate a clear boundary for digital wellbeing: while “cyber-social relations” (Scheibe et al., 2022), live singing provide essential psychological triage and bonding capital, they should be conceptualized as complementary to, rather than a holistic substitute for, offline cross-cultural adaptation. In addition, because the data are cross-sectional, the ordering of mediators reflects a theoretical assumption, not an established temporal sequence. The same data could be compatible with alternative orderings. It is plausible, for instance, that individuals who already perceive strong social support are more inclined to feel socially connected during interactions, or that both variables are jointly influenced by the same antecedent conditions. Longitudinal or experimental designs would be needed to evaluate whether this sequential ordering reflects a directional process.

The finding that fear of missing out attenuated the associations of live interactive singing behavior with social connection, perceived social support, and loneliness is consistent with a growing body of evidence that the psychological correlates of active digital participation are contingent on the user’s dispositional orientation. These FoMO findings warrant interpretation through the specific characteristics of the present sample. Participants were Mainland Chinese students studying abroad in 11 host countries (most prominently the United States, the United Kingdom, Canada, and Australia), with a mean of 20.24 months in the host country (SD = 14.23) and a substantial subgroup in the early-adjustment window (median = 18 months).

For this population, FoMO is unlikely to function as it does for stationary domestic users of social media. Geographic separation from home-country social circles renders the very social world that FoMO targets—the friendships, family gatherings, and informal networks left behind—both salient and inaccessible. When this population logs onto native-language live-streaming platforms, they enter a digital environment that simultaneously displays the home-country social life they have lost access to and offers a participatory channel through which it might be partially regained. FoMO, in this context, is not merely an anxious orientation toward online content; it represents a specific digital manifestation of acculturative stress associated with the distance between the participant and the home-culture social field (Jarrar and Nweke, 2025). Within this “digital diaspora,” the constant visibility of the left-behind social world may be linked to painful upward social comparisons, where individuals potentially experience a supportive environment as a source of digital stress (Kianpour et al., 2025; Putri et al., 2026).

This contextual reading helps explain why FoMO’s moderating role was particularly pronounced for the direct and support-only pathways, which depend on the relational quality of individual interactions, while the serial pathway—mediated through the cumulative experience of belonging—was relatively more resistant. For internationally displaced users, anxiety-driven and comparison-heavy engagement may be negatively associated with immediate interactional payoffs even as longer-arc relational accumulation continues, slowly, to develop. In support of this mechanism, O’Day and Heimberg (2021) identified fear of missing out and rumination as key variables linked to problematic social media use among socially anxious individuals (Dempsey et al., 2019), and Yang (2016) similarly found that social comparison—a process central to FoMO—moderated the association between social media use and loneliness.

The present study extends this line of work by showing that fear of missing out was associated with weaker associations across multiple pathways within a single integrated model, rather than moderating an isolated bivariate relationship. The nonsignificance of both the direct association and the indirect pathway through perceived social support at high fear of missing out, together with the attenuation of the serial pathway, indicates that fear of missing out weakened several, though not all, of the associations linking live interactive singing behavior to lower loneliness. The Johnson-Neyman analysis revealed that the direct pathway was the most susceptible to attenuation, losing significance for approximately one-third of the sample, while the pathway to social connection was comparatively more stable.

Qualitative evidence from research on mobile populations offers a parallel observation. Miguel et al. (2025) found that anxiety-driven pursuit of constant connectivity among highly mobile individuals was associated with shallow encounters that reinforced rather than alleviated perceived loneliness. The present quantitative findings are consistent with this observation: at higher levels of fear of missing out, the associations between live interactive singing behavior and relational outcomes were significantly weaker, suggesting that dispositional anxiety may moderate the relational yield of digital engagement.

### 5.3 Theoretical contributions

This study offers three contributions to the literature on digital social interaction and psychological wellbeing. First, the findings contribute to the refinement of the active use hypothesis in media psychology. The prevailing framework categorizes social media behaviors as active or passive, with the assumption that active use is inherently psychologically beneficial (Burke et al., 2011; Verduyn et al., 2017). However, meta-analytic evidence indicates that this dichotomy is too coarse and that substantial heterogeneity exists (Godard and Holtzman, 2024; Kross et al., 2021; Valkenburg et al., 2022). The present study provides evidence that the same active behavior—live interactive singing—was associated with different patterns of loneliness depending on the level of fear of missing out. This finding is compatible with the extended active-passive model (Verduyn et al., 2022), demonstrating that the psychological correlates of active participation depend jointly on the functional characteristics of the behavior (synchronous, directed, high in social presence; Hall, 2018) and the dispositional orientation of the user.

Second, the study provides evidence for a sequential arrangement of social connection and perceived social support in the association between digital engagement and loneliness. By showing that a model specifying these constructs as ordered stages fits the data, this study advances beyond prior work that treated social connection and social support as interchangeable or parallel mediators (e.g., Zhang et al., 2021). The sequential specification theoretically distinguishes momentary affective states from stable cognitive appraisals, identifying the link between situational belonging and stable support perception as a critical maturation component of the model. This finding is consistent with social presence theory’s focus on immediate experiential connections (Biocca et al., 2003) and social capital theory’s proposition that relational trust must accumulate before being linked to the perception of available resources (Lin, 1999).

Third, the sensitivity analysis provides preliminary evidence that the moderated mediation pattern was broadly consistent across two academic-level subgroups, which offers some confidence in the stability of the associations within this sample. Rather than being an artifact of a specific acculturative phase, the findings suggest a fundamental psychological mechanism at play. The minor differences between subgroups not only highlight the importance of considering statistical power when interpreting subgroup variations, but also empirically respond to calls from the Differential Susceptibility to Media Effects Model (Valkenburg and Peter, 2013) for research specifying “when, how, and for whom” digital participation is linked to psychological outcomes (Kross et al., 2021; Verduyn et al., 2022).

### 5.4 Practical considerations

Although the cross-sectional design precludes prescriptive recommendations, the present findings suggest two evidence-informed directions that warrant prospective testing. Because international students face language and cultural barriers that limit the feasibility of host-institution-organized programming, both directions are addressed to live-streaming platforms, which already operate in the participants’ native language.

First, platform-organized recurring co-singing events targeted at internationally mobile users could test whether structured participation produces detectable changes in relational outcomes. Native-language platforms (e.g., Bilibili Live, NetEase CC, KuGou Live) already host themed events and could partner with diaspora-oriented streamers to schedule recurring sessions—two per week of 60 to 90 min—organized into stable cohorts that persist across multiple sessions. The 1–2 sessions/week target reflects the engagement regime in which the present associations were observed (sample M = 3.18, modal “sometimes”); higher frequencies are unsupported by the data and theoretically risky given the FoMO findings. Cohort continuity follows from the present finding that relational benefits accumulate through sustained interaction with the same community rather than through diffuse encounters. Brief in-platform messaging on anxiety-driven engagement, delivered alongside event onboarding, would address the boundary condition identified here. Outcomes can be evaluated through opt-in pre–post surveys embedded in the platform interface.

Second, platform feature design could test three directions consistent with the present model: recurring co-singing rooms with stable membership and follow-streak indicators, which support the cumulative interactional history through which connection developed into perceived support; streamer-side prompts for vocal acknowledgment of co-participating viewers, mapped directly onto the streamer-recognition behavior indexed by item LISB4; and engagement-pattern-aware nudges recommending breaks or consolidation when usage profiles—rapid serial-room sampling, brief high-frequency sessions, late-night spikes—suggest anxiety-driven participation. Each can be evaluated through within-platform A/B trials.

### 5.5 Limitations

Several limitations must be acknowledged. First, and most fundamentally, the cross-sectional design precludes causal or temporal inference. Although the moderated serial mediation model implies a directional sequence, the data are equally consistent with reverse pathways: loneliness may drive individuals toward live interactive singing as a compensatory behavior, social connection and perceived social support may shape the propensity to engage in co-singing rather than the reverse, or unmeasured third variables may account for the observed associations. The statistical ordering of the mediation model reflects theoretical assumptions about how relational processes unfold, not established temporal precedence.

Second, the sample is restricted to Mainland Chinese students studying abroad who use native-language live-streaming platforms and have engaged in at least four live interactive singing sessions in the past month. This scope was deliberate—it isolates a population for whom culturally congruent digital singing is a meaningful psychological resource—but it limits generalizability in three respects. The findings cannot be assumed to extend to international students from other origins, whose live-streaming ecosystems and cultural relationships to vocal performance differ; they cannot be extrapolated to students who do not use native-language platforms, who may rely on different compensatory strategies; and they cannot speak to non-users of live interactive singing. Recruitment relied on a market research firm’s panel supplemented by referral sampling, which constitutes a non-probability strategy. Although the panel was screened against three eligibility criteria, panel-based sampling tends to over-represent digitally active and incentive-responsive segments, and the snowball component introduces network homogeneity. Together, these features mean that the findings should be read as applying to engaged users within a single nationality–platform–behavior configuration, not to international students broadly.

Third, the live interactive singing behavior scale was newly adapted for this study. Although three items were drawn from the validated Streamer–Viewer Interaction dimension of Taieb et al.'s (2026) cross-culturally invariant OLSI scale and two items from Ozimek et al.'s (2023) SMAQ, and although the present-sample CFA, composite reliability, and discriminant validity evidence (§4.1) supports the unidimensional structure, the AVE for the scale (0.47) fell marginally below the conventional 0.50 threshold. Further psychometric work across independent samples is needed before the adapted scale can be considered established.

Fourth, loneliness was measured as a general psychological state rather than differentiated into its emotional and social subtypes. Emotional loneliness arises from the absence of an intimate attachment figure; social loneliness arises from a deficit in one’s broader social network. The two may respond differently to live interactive singing: the behavior’s communal-belonging affordances are theoretically well-suited to reducing social loneliness, but its capacity to substitute for intimate ties is more limited. By aggregating the two, the present study likely understates the differential pathway through which live interactive singing operates. Future research using subtype-differentiated measures is needed to recover this distinction.

Fifth, all focal constructs were measured via participant self-report. Although Harman’s single-factor test and the excellent five-factor measurement model fit (§4.1) suggest that common method variance does not dominate the data, retrospective self-reports of singing behavior, social connection, and perceived social support remain susceptible to recall bias, social desirability, and subjective interpretation. Platform-logged behavioral data—frequency of co-singing sessions, duration, identity of co-participants—would provide a more objective behavioral indicator and should be incorporated in future work where ethics-board approval and platform partnership permit.

Sixth, the present design focuses on a single interactive feature (live interactive singing) within a single digital environment (native-language Chinese live-streaming platforms). While this specificity is a strength in terms of measurement precision, it limits direct comparison with other forms of active live-stream participation (e.g., danmu commenting, virtual gift-giving, watch-along streaming) and with platforms operating under different cultural and architectural conventions (e.g., Twitch, YouTube Live, AfreecaTV).

Seventh, the effect sizes observed, although statistically robust at the present sample size, were modest in absolute magnitude (*R*^2^ = 0.17 for the social connection equation, 0.24 for the perceived social support equation, 0.38 for the loneliness equation). Live interactive singing should therefore be understood as one contributing relational resource among many for international students experiencing loneliness, not as a singular intervention point.

Finally, the academic-level variable was specified at the binary undergraduate-versus-graduate level, precluding finer distinctions among graduate populations (master’s, doctoral, postdoctoral). Future research with larger and more granular samples could examine whether the observed associations vary across these subgroups.

### 5.6 Future research directions

The present findings open four concrete directions for future inquiry, each tied to a specific limitation above. Longitudinal and intensive-longitudinal designs are needed to establish temporal precedence. A 14-day ecological momentary assessment protocol, in which international students report live interactive singing participation, social connection, perceived support, and loneliness in evening diary entries, would permit within-person tests of whether singing on day t predicts changes in social connection on day t + 1 and changes in loneliness on day *t* + 7. Such designs would also allow tests of the FoMO moderation at the within-person level: do anxiety-elevated days yield smaller relational payoffs from singing within the same individual? Two-wave panel designs with three-month spacing could complement EMA work by capturing the slower trajectory through which situational connection accumulates into stable support perceptions.

Experimental and quasi-experimental designs could provide stronger directional evidence. A pre–post field study comparing structured live interactive singing sessions (organized through international student associations) against passive live-stream viewing or a no-treatment control would test whether singing participation produces detectable changes in loneliness over a four-to-eight-week window. Random assignment within international student cohorts is feasible at the program level.

Multi-method behavioral measurement should integrate platform-logged engagement data (with appropriate consent and ethics review) alongside self-report. Combining objective frequency and duration measures, network indicators (whether the same streamer/community is repeatedly engaged), peer-report measures of perceived support, and physiological indicators of social presence (e.g., synchrony of vocal pitch contours) would substantially strengthen measurement validity and reduce reliance on shared method variance.

Cross-cultural and cross-platform comparative research should determine whether the observed associations are general or culturally specific. Three contrasts are particularly informative: Mainland Chinese students using native-language platforms versus international students from other origins using their own native-language platforms (Korean, Spanish-speaking, Arabic-speaking, etc.); native-language platform users versus host-language platform users within the same population; and structurally singing-centric platforms (e.g., Chinese live-streaming) versus structurally chat-centric or game-centric platforms (e.g., Twitch). Such comparisons would distinguish the contribution of cultural-linguistic congruence from the contribution of synchronous vocal interaction itself.

Finally, the practical implications identified here could be tested through intervention research. Collaborations among researchers, university international student services, and live-streaming platforms could develop and evaluate structured co-singing programs, including psychoeducational components on FoMO-driven engagement, and could test whether platform features that promote sustained interaction with a consistent community (rather than diffuse one-off encounters) enhance the relational correlates of participation.

## 6 Conclusion

This study found that live interactive singing behavior was negatively associated with loneliness among international students, and that this association was statistically consistent with indirect pathways through social connection and perceived social support. Fear of missing out moderated these associations, such that the negative link between live interactive singing behavior and loneliness was weaker at higher levels of fear of missing out. The pattern was broadly replicated across undergraduate and graduate subgroups. These findings extend the literature on online social interaction and wellbeing by identifying live interactive singing as a specific participatory behavior associated with lower loneliness in a population facing elevated loneliness risk. However, because the data are cross-sectional and self-reported, the findings identify associative patterns rather than directional or causal relationships. Longitudinal and experimental research is needed to evaluate whether live interactive singing behavior may play a role in addressing loneliness among international students.

## Data Availability

The raw data supporting the conclusions of this article will be made available by the authors, without undue reservation.
